# SARS-CoV-2 Whole-Genome Sequencing Using Oxford Nanopore Technology for Variant Monitoring in Wastewaters

**DOI:** 10.3389/fmicb.2022.889811

**Published:** 2022-06-09

**Authors:** Laure Barbé, Julien Schaeffer, Alban Besnard, Sarah Jousse, Sébastien Wurtzer, Laurent Moulin, OBEPINE Consortium, Jean-Luc Bailly, Françoise S. Le Guyader, Marion Desdouits

**Affiliations:** (Laboratoire Microorganismes, Génome et Environnement, LMGE, UMR 6023 CNRS-Université Clermont Auvergne, Clermont-Ferrand, France), (Laboratoire de Chimie Physique et Microbiologie pour les Matériaux et l’Environnement, LCPME, UMR 7564 CNRS-Université de Lorraine, Nancy, France), (Ifremer Nantes, France), (Institut Carnot Smiles, Sorbonne Université, Laboratoire Jacques-Louis Lions, UMR 7598 and Institut Universitaire de France), (Sorbonne Université, INSERM, Centre de Recherche Saint-Antoine, 75012, Paris, France), (Sorbonne Université, CNRS, EPHE, UMR 7619 Metis, Paris, France, e-LTER Zone Atelier Seine), (Laboratoire R&D, Eau de Paris, France), (Val de Grâce, Unité de Virologie de l’IRBA. Institut de Recherche Biomédicale des Armées, Brétigny, France), (Laboratoire R&D, Eau de Paris, France); ^1^Laboratoire de Microbiologie (LSEM, Unité MASAE), IFREMER, Nantes, France; ^2^R&D Laboratory, DRDQE, Eau de Paris, Ivry-sur-Seine, France

**Keywords:** SARS-CoV-2, variant of concern, wastewater-based epidemiology, next-generation sequencing, Oxford Nanopore Technology, sewage, ARTIC

## Abstract

Since the beginning of the Coronavirus Disease-19 (COVID-19) pandemic, multiple Severe Acute Respiratory Syndrome Coronavirus 2 (SARS-CoV-2) mutations have been reported and led to the emergence of variants of concern (VOC) with increased transmissibility, virulence or immune escape. In parallel, the observation of viral fecal shedding led to the quantification of SARS-CoV-2 genomes in wastewater, providing information about the dynamics of SARS-CoV-2 infections within a population including symptomatic and asymptomatic individuals. Here, we aimed to adapt a sequencing technique initially designed for clinical samples to apply it to the challenging and mixed wastewater matrix, and hence identify the circulation of VOC at the community level. Composite raw sewage sampled over 24 h in two wastewater-treatment plants (WWTPs) from a city in western France were collected weekly and SARS-CoV-2 quantified by RT-PCR. Samples collected between October 2020 and May 2021 were submitted to whole-genome sequencing (WGS) using the primers and protocol published by the ARTIC Network and a MinION Mk1C sequencer (Oxford Nanopore Technologies, Oxford, United Kingdom). The protocol was adapted to allow near-full genome coverage from sewage samples, starting from ∼5% to reach ∼90% at depth 30. This enabled us to detect multiple single-nucleotide variant (SNV) and assess the circulation of the SARS-CoV-2 VOC Alpha, Beta, Gamma, and Delta. Retrospective analysis of sewage samples shed light on the emergence of the Alpha VOC with detection of first co-occurring signature mutations in mid-November 2020 to reach predominance of this variant in early February 2021. In parallel, a mutation-specific qRT-PCR assay confirmed the spread of the Alpha VOC but detected it later than WGS. Altogether, these data show that SARS-CoV-2 sequencing in sewage can be used for early detection of an emerging VOC in a population and confirm its ability to track shifts in variant predominance.

## Introduction

Shedding of SARS-CoV-2 *via* human feces results in the presence of viral genetic material in human sewage, thus allowing wastewater-based epidemiology (WBE). WBE relies on the fact that anytime a stable molecule or micro-organism is excreted by humans and later drained into wastewater, the original concentration excreted by the serviced population can be inferred from sewage sample analysis (Madoux-Humery et al., 2016; Mao et al., 2020; Polo et al., 2020). Prior to the COVID-19 pandemic, other members of the *Coronaviridae* family had already been identified in wastewater (Wang et al., 2005; Bibby and Peccia, 2013) but not for epidemiological purpose. This approach is particularly interesting as it provides additional information about the dynamics of SARS-CoV-2 infections at the community level. Indeed, it includes symptomatic but also asymptomatic individuals which can represent between 10.1 and 23.0% of the infected population for SARS-CoV-2 and largely contribute to the silent spread of the disease (He et al., 2021). WBE has been used since the beginning of the COVID-19 pandemic in several countries and numerous studies demonstrated temporal correlations between SARS-CoV-2 RNA titers in sewage and the number of human cases in the corresponding population (Ahmed et al., 2020; Medema et al., 2020; Wu et al., 2020, 2022; Wurtzer et al., 2020; Amereh et al., 2021). These results indicate that monitoring of wastewater can serve as an early warning tool to inform public health authorities (Farkas et al., 2020). This approach, previously used for human enteric viruses (Miura et al., 2016), is innovative concerning a respiratory, enveloped virus.

Most of these WBE studies used quantitative reverse transcription-PCR to detect and estimate the viral concentration. This technique is sensitive and specific but it gives little information on the genomic sequence. With the increase of SARS-CoV-2 genetic diversity and hence emergence of new lineages, genome analysis is essential to monitor evolution, transmission, and spread of variants of the virus. It also implies that additional techniques such as sequencing and/or mutation-specific SARS-CoV-2 RT-PCR tests should be considered for WBE. Since Chinese health authorities first shared the SARS-CoV-2 complete genome sequence, >8,800,000 genomes have been sequenced as of March 2022, mostly from clinical samples. This worldwide effort in SARS-CoV-2 whole genome sequencing (WGS) was made possible, among many factors, by the design of multiplex PCR panels such as those shared by the ARTIC Network (Tyson et al., 2020). Because of the virus genetic diversity, these data allowed to describe groups and associate them with geographic and temporal pattern of virus spread. This diversity is described by the Nextstrain project^1^ which divides SARS-CoV-2 into 25 major clades (19A-B, 20A-20J, and 21A-M) based on high prevalence, signature mutations and geographic spread (Hadfield et al., 2018).

The wastewater matrix poses several challenges for sequencing of SARS-CoV-2: (1) the viral load is low compared to most clinical samples, (2) the genetic diversity represents a mix of strains infecting many different people, (3) a high proportion of the viral genomes is unprotected and likely fragmented (Wurtzer et al., 2021b), precluding the amplification and sequencing of whole genomes from single RNA molecules, (4) the matrix itself contains a high diversity of other genetic materials and chemicals, some known as PCR inhibitors. To circumvent these issues, SARS-CoV-2 whole-genome sequencing (WGS) protocols need to be adapted at all steps—RNA extraction, cDNA synthesis, genome amplification, library preparation, and bioinformatics analysis. To date, several studies have demonstrated that SARS-CoV-2 RNA sequencing from wastewater could help to understand the city- or country-scale circulation of SARS-CoV-2 variants (Nemudryi et al., 2020; Agrawal et al., 2021; Bar-Or et al., 2021; Crits-Christoph et al., 2021; Fontenele et al., 2021; Izquierdo-Lara et al., 2021; Jahn et al., 2021; Prado et al., 2021; Rouchka et al., 2021; Rubio-Acero et al., 2021; Wilton et al., 2021). SARS-CoV-2 WGS in sewage was conducted using multiplex PCR panels combined mostly with Illumina sequencing (Ai et al., 2021; Bar-Or et al., 2021; Fontenele et al., 2021; Hillary et al., 2021; Izquierdo-Lara et al., 2021; Jahn et al., 2021; Mondal et al., 2021; Prado et al., 2021; Rouchka et al., 2021; Rubio-Acero et al., 2021; Wurtz et al., 2021), and more rarely with Oxford Nanopore Technology (Nemudryi et al., 2020; Izquierdo-Lara et al., 2021; Rios et al., 2021). Here, we aimed to adapt a sequencing technique using the widely used and frequently updated ARTIC-400 panel of primers (Tyson et al., 2020) and Oxford Nanopore Technology (ONT), initially designed for clinical samples, to apply it to the challenging wastewater matrix. This technique enabled to observe single nucleotide variants specific of the Alpha and Beta variants of concern (VOC) and to detect the Alpha VOC at the community level in a French city, earlier than using a variant-specific quantitative RT-PCR assay.

## Materials and Methods

### Virus Stocks and Cell Lines

Mengovirus (MgV) strain pMC0 (kindly provided by A. Bosch, University of Barcelona, Spain) was propagated in HeLa cells as previously described (Martin et al., 1996).

### Sample Collection and Extraction

Untreated wastewater (raw sewage) samples were collected on a weekly basis at two wastewater treatment plants (WWTPs), WWTP1 and WWTP2, serving a total of 644,000 inhabitants (446,000 and 198,000, respectively) in the same city, between October 2020 and May 2021. For this study, 38 samples from WWTP1 and 38 from WWTP2 were used for sequencing. In addition, four samples used for method adaptation were collected in WWTP3, serving 22,000 inhabitants in a smaller city, between March and April 2021. All samples are listed in Supplementary Table 1. The 24-h flow-dependent composite samples (1–2 L) were collected in the morning, transported on ice to the laboratory and stored at 4°C for 0–2 days before the first analysis consisting in SARS-CoV-2 RNA quantification. For the retrospective part of our study, wastewater samples were analyzed after storage at −20°C for up to >1 year and thawed by overnight incubation at 4°C. All samples were homogenized and a subsample of 11 mL was ultracentrifugated for 1 h at 100,000 × g as described in Wurtzer et al. (2020). Pellet was resuspended in 500 μL of Phosphate-Buffered Saline (PBS). Nucleic acids (NAs) were subsequently extracted by using the NucliSens kit and the NucliSens miniMAG purification system (bioMérieux, Marcy L’Etoile, France) following the manufacturer’s instructions, with 2 mL lysis buffer, 50 μL magnetic silica and eluted in 100 μL elution buffer. Extracted NAs were further cleaned up using the OneStep PCR inhibitor removal kit (Zymo Research, Irvine, CA, United States), following the manufacturer’s instructions.

### Process Control

Mengovirus, a murine picornavirus, was used as a process control for nucleic acid (NA) extraction. MgV or other non-enveloped viruses were used as process control for SARS-CoV-2 WBE by other teams previously (Barril et al., 2021; Alamin et al., 2022; Brnić et al., 2022). Here it was considered adequate as our concentration step relies on ultracentrifugation, which is efficient on both enveloped and non-enveloped virions, and early tests showed similar efficiencies using a porcine coronavirus (data not shown). Briefly, 100 μL of MgV solution (10^6^ cRNA) were added to each 11 mL wastewater subsample prior to ultracentrifugation and each series of NA extractions included an extraction control in the form of 100 μL of pure MgV solution. MgV concentration in NAs extracted from sewage samples were compared to that of the extraction control to calculate the extraction efficiency of each sample (Supplementary Table 1).

### Quantitative One-Step Reverse Transcription and PCR and Genome Copy Quantification

The Ultrasens kit (Thermo Fisher Scientific, Illkirch-Graffenstaden, France) was used for all quantitative one-step reverse transcription and PCR (qRT-PCR) assays, following the manufacturer’s instructions, using an Aria Mx or MxP3000 real-time PCR system (Agilent Technologies, Santa Clara, United States) (Desdouits et al., 2021). The MgV qRT-PCR assay was carried out as previously described (Le Guyader et al., 2009) on 5 μl of pure NA extract and of a 10-fold dilution, to assess the presence of PCR inhibitors. After verification of extraction efficiency using MgV, 5 μl of pure NA extract in triplicate were screened for SARS-CoV-2 using two sets of primers and probes: IP4, targeting the polymerase gene, used to quantify SARS-CoV-2, and SΔ69/70, targeting the 69/70 HV deletion on the spike gene, designed to assess and quantify the Alpha VOC (Supplementary Table 2; Wurtzer et al., 2021a). Thermal profile was adapted to comply with the one-step qRT-PCR kit requirements: reverse-transcription for 15 min at 55°C, first denaturation and *Taq* polymerase activation for 5 min at 95°C, and 45 cycles of denaturation (94°C, 15 s), annealing (58°C, 30 s) and extension (65°C, 30 s) followed by fluorescence acquisition. For quantification, 5-point standard curves in duplicate were made by serial dilution of a SARS-CoV-2 RNA transcript (CNR des virus respiratoires, Pasteur Institute, Paris, France) for the IP4 PCR, and of a NA extracted from B.1.1.7 strain for the Δ69/70 PCR (Centre de Recherche en Transplantation et Immunologie, UMR1064, ITUN, Nantes, France). Good laboratory practices were observed throughout the analysis process, with dedicated separate rooms for wastewater processing, NA extraction, preparation of PCR mixtures, template addition, positive controls addition, and amplification. No-template controls were included in all qRT-PCR assays and proved always negative.

### cDNA Generation

Reverse transcription was performed with 15 μL of NAs extracted from SARS-CoV-2 positive wastewater samples using SuperScript II Reverse Transcriptase (Thermo Fisher Scientific, Illkirch-Graffenstaden, France) following a modified protocol (Strubbia et al., 2019). Briefly, 15 μL of RNA, either freshly extracted and stored at 4°C for up to 2 weeks, or stored frozen at −20°C for up to 10 months and thawed at room temperature, were mixed with 4.6 μL random hexamers (Themo Fisher Scientific) in presence of 3 μL 10× ligase buffer (Thermo Fisher Scientific, Illkirch-Graffenstaden, France) and 2.4 μL 100 mM MgCl_2_. The reaction was incubated at room temperature for 2 min and the following components were added to the mix: 2 μL 10× ligase buffer, 1 μL SuperScript II Reverse Transcriptase, 1 μL dNTPs at 25 mM each, 1 μL DTT and 15 μL nuclease-free water. Then, the reaction was incubated for 90 min at 37°C and for 20 min at 70°C.

### Library Preparation and Sequencing

**FIGURE 1:**
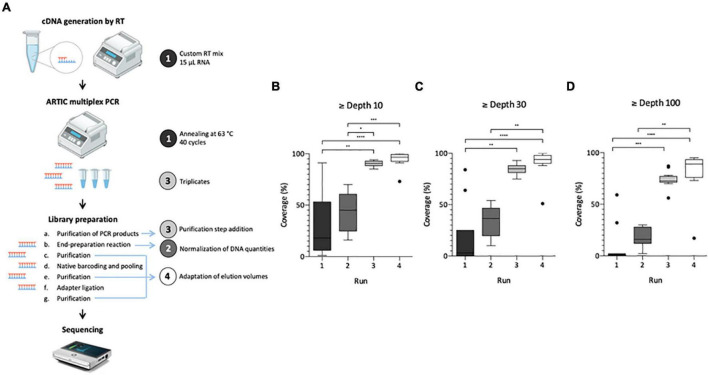
Adaptation process of SARS-CoV-2 genome sequencing from wastewater samples. Left panel illustrates the protocol summary **(A)** with adaptation lines 1–4 depicted by a colored circle, same colors are used for box plot panels **(B–D)**. Box Plot (Tukey whiskers) of SARS-CoV-2 genome coverage percentages obtained per run of sequencing on raw wastewater samples at depth 10 **(B)**, 30 **(C)**, and 100 **(D)** during the adaptation process of the ARTIC protocol. Adaptation lines were for Run 1: cDNA synthesis (15 μ1 RNA extract, random hexamers and SuperScript II reverse transcriptase), ARTIC multiplex PCR (annealing at 63°C, 40 cycles); Run 2: library preparation (normalization of initial DNA quantities); Run 3: ARTIC multiplex PCR (triplicates for each pool), library preparation (addition of an initial purification step of PCR products); Run 4: library preparation (addition of a purification step between the end-preparation and the barcoding reactions, adaptation of elution volumes to maximize recovery). Kruskal-Wallis test followed by Dunn’s multiple comparisons test were used to compare groups (^****^p < 0.0001, ^***^p < 0.001, ^**^p < 0.01, *p < 0.05 and not significant if no indication on the plot) Panel A is adapted from Hourdel et al. (2020).

Generated cDNA were used as a template for SARS-CoV-2-specific multiplex PCR. The ARTIC v3 Panel (designed by Josh Quick, University of Birmingham and marketed by Integrated DNA Technologies, United States) consists of 98 amplicons of approximatively 400 bp in length, spanning the entire genome (Tyson et al., 2020). These primers were used in two PCR pools according to the ARTIC network’s instructions (ncov-2019-sequencing-protocol-v3-locost). PCR were performed in triplicate for each pool using 8.5 μL cDNA as template, under the following conditions: heat activation for 30 s at 98°C and 40 cycles of denaturation (95°C, 15 s), annealing and extension (63°C, 5 min). Amplicons for the same sample were pooled and used as a template for library synthesis following the ARTIC nCoV-2019 sequencing protocol v3 (ncov-2019-sequencing-protocol-v3-locost). A few modifications were performed as described below (Figure 1A). Pooled amplicons were purified with 0.8× SPRIselect beads (Beckman Coulter, Fullerton, CA, United States) following the manufacturer’s instructions and Fullerton, CA eluted in 10 μL nuclease-free water. Concentrations were measured by fluorescence in a Qubit 3 (Thermo Fisher Scientific, Illkirch-Graffenstaden, France) using the Qubit dsDNA HS Assay Kit (Thermo Fisher Scientific, Illkirch-Graffenstaden, France) following the manufacturer’s instructions. Purified amplicons were diluted with nuclease-free water in 8.3 μL total, using the sample having the lowest concentration to define the quantity added for each sample (150–400 ng). A purification step was added following the end-preparation reaction using 1× SPRIselect beads and resuspending in 5 μL nuclease-free water. Then, 3.75 μL of the purified end-preparation reaction mixture were mixed with barcodes accordingly using the Oxford Nanopore native barcoding kit (NBD-104, Oxford Nanopore Technologies, Oxford, United Kingdom). The last purified product was eluted in 13 μL of elution buffer. Finally, the library was loaded on a R9.4.1 flow cell placed onto a MinION Mk1C sequencer for a 14–18 h run. Any difference between the described method and the ARTIC nCoV-2019 sequencing protocol v3 is part of the adaptation process (Figure 1A).

### SARS-CoV-2 Sequence Analysis

After the sequencing runs, fast5 data files were base-called using Guppy (version 4.3.4, Oxford Nanopore Technologies, Oxford, United Kingdom) to generate fastq files (available at https://data-dataref.ifremer.fr/bioinfo/ifremer/obepine/lsem/data/dna-sequence-raw/). Successfully base-called reads were further analyzed following the ARTIC nCoV-2019 pipeline version 1.2.1,^2^ which included demultiplexing, read filtering, primers and barcode trimming. The resulting alignment file was used for single nucleotide variants (SNVs) calling using LoFreq version 2.1.5 with minimum base quality of 20 and 20× coverage, relative to Wuhan-Hu-1/2019 reference genome (GenBank: MN908947.3). Short indels calling was also performed using Lofreq after a preprocessing step to insert indel qualities. Samtools was used to read alignment files and an Awk-based script enabled to extract genome coverage percentages at depth 10, 30, and 100. Samtools also allowed the extraction of mean genome coverage across the distinct amplicons trimmed for primer and overlapping sequences, for each sample. For VOC analysis, we excluded samples with depth 30 coverage <70%. On the basis of previous studies (Martin et al., 2020; Izquierdo-Lara et al., 2021), single nucleotide variants (SNVs) and indels with coverage < 30, average quality < 30, frequency < 5%, and homopolymer run > 4 (for indels only) were excluded. The detected SNVs were filtered by position and compared with the signature mutations for alpha, beta, gamma and delta VOC described in https://nextstrain.org and listed in Supplementary Table 3. Additional details on sequencing runs are available in the Table 1.

**TABLE 1:** Additional details on sequencing runs.

#	Objective*	Total samples	Library quantity (ng)	Total mapped reads	Median depth 30 coverage (%)
1	Adaptation	12	50	32,293	3
2	Adaptation	8	38	39,530	36
3	Adaptation	12	122	295,224	85
4	Adaptation and prospective	10	346	288,608	94
5	Retrospective	12	317	285,529	90
6	Retrospective	12	222	315,498	88
7	Retrospective	12	106	144,754	68
8	Retrospective	9	194	112,553	55

*Objective of the sequencing run: protocol adaptation for WW samples (runs 1–4), prospective sequencing of fresh WW samples (run 4), retrospective sequencing of stored WW samples (runs 5 to 8).

### Statistical Analysis

GraphPad Prism v 9.0.0 (GraphPad Software, San Diego, CA, United States) was used for data representation and statistical analysis. Comparisons for evaluation of the impact of each adaptation line during the adaptation process and freezing on the adapted sequencing protocol were performed using Kruskal-Wallis test followed by Dunn’s multiple comparisons test. Correlations between SARS-CoV-2 genomic concentrations and genome coverage for all samples and each group individually (i.e., fresh or frozen RNA and frozen wastewater) were assessed using the Spearman non-parametric test. Differences were considered statistically significant when *p* < 0.05.

## Results

### Sequencing Protocol Adaptation for Wastewater Samples

The first aim of this study was to adapt the ARTIC V3 Lo-cost protocol, initially designed for clinical samples, in order to use it for SARS-CoV-2 WGS in raw wastewater samples. Four sequencing runs were needed to achieve this objective as illustrated on Figure 1. For each run, modifications made to the original protocol are indicated on Figure 1A. Results obtained for the first run were heterogeneous and median coverages were low (18, 3 and 0% at depth 10, 30, and 100, respectively) (Figures 1B–D), confirming the need to adapt the initial protocol to sewage samples. Normalizing the DNA quantity for each sample enabled to reduce genome coverage disparity in run 2 and increase median coverage (45, 37, and 16%, respectively) albeit not significant (Figures 1B–D). For the third run, pooling triplicate ARTIC PCR and purifying the PCR products allowed to significantly improve these results with medians of 91, 85, and 73%, at depth 10, 30, and 100, respectively (Figures 1B–D). Finally, the adaptation of elution volumes enabled further improvement of the process in run 4 with medians of 97, 94, and 89% at depth 10, 30, and 100, respectively (Figures 1B–D). Altogether, these modifications enabled the implementation of an adapted protocol suitable for SARS-CoV-2 genome sequencing in wastewater samples.

### Impact of Freezing and SARS-CoV-2 Concentration

**FIGURE 2:**
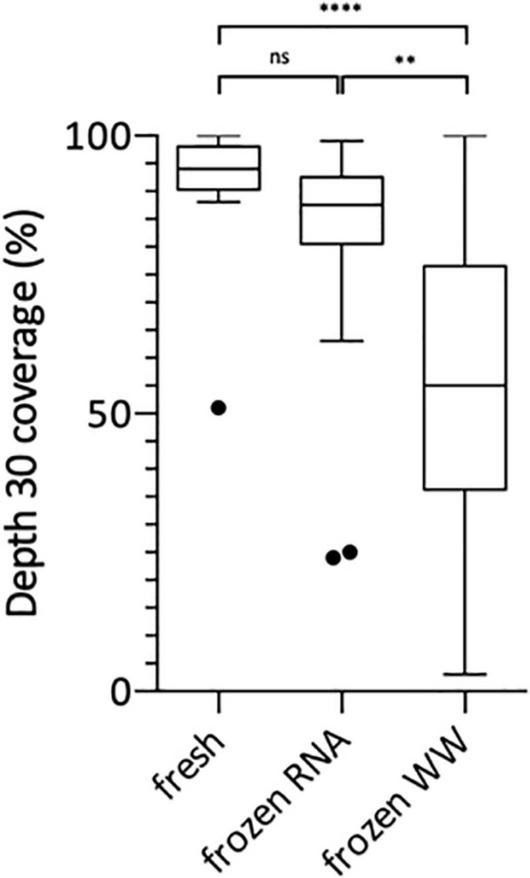
Box Plot of SARS-CoV-2 genome coverage percentages obtained at depth 30 using the previously adapted method and starting from freshly prepared RNA, frozen RNA and frozen wastewater (WW). Kruskal-Wallis test followed by Dunn’s multiple comparisons test were used to compare groups (^****^p < 0.0001, ^**^p < 0.01, ns: not significant).

**FIGURE 3:**
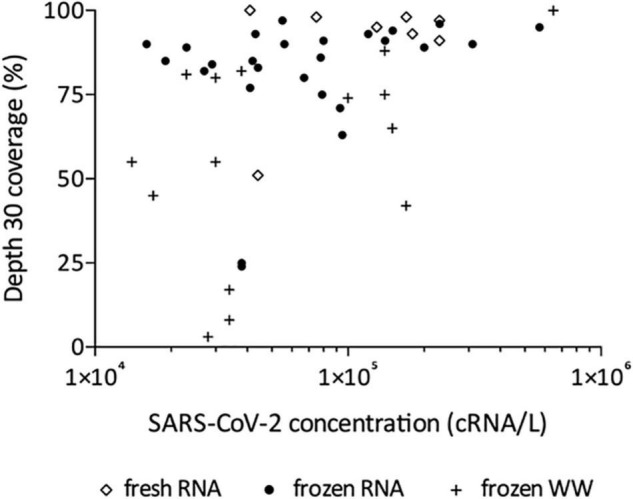
Effect of SARS-CoV-2 concentration on depth 30 genome coverage using the adapted method and starting from freshly prepared RNA, frozen RNA and frozen wastewater (WW). Spearman test was used to test correlation between the two parameters for all samples (*p* = 0.0004, *r* = 0.4881) and each group individually: frozen RNA samples (*p* = 0.0349, *r* = 0.4153), fresh RNA samples (*r* = 0.3253, *p* > 0.05), and frozen WW samples (*r* = 0.3522, *p* > 0.05).

Following this technical adaptation, a retrospective analysis was conducted using samples stored as frozen NA extracts or raw wastewater. This allowed us to compare the sequencing depth and coverage reached with fresh and frozen material (Figure 2 and Supplementary Table 1). Best coverage percentages were obtained starting from freshly prepared RNA extracts with a median of 94% ranging from 51 to 100% at depth 30 (Figure 2). When using frozen RNA extracts as initial matrix for cDNA synthesis, genome coverage percentages were reduced to 88% and distribution seemed more heterogeneous ranging from 24 to 99%, but this difference was not statistically significant (Figure 2). The use of frozen wastewater samples had a significant impact causing a strong coverage decrease and an increase in distribution heterogeneity (*p* < 0.0001 and *p* < 0.01 when compared to fresh and frozen RNA respectively), with a median of 55% ranging from 3 to 100% (Figure 2). Then, we studied the impact of SARS-CoV-2 concentration, measured by qRT-PCR, on the depth 30 genome coverage (Figure 3 and Supplementary Table 1). A weak correlation was observed between the two parameters when considering all samples (*p* = 0.0004, *r* = 0.4881) or the frozen RNA samples only (*p* = 0.0349, *r* = 0.4153) but not the fresh or frozen WW samples (*r* = 0.3253 and *r* = 0.3522, respectively, *p* > 0.05 for both). There was also no correlation between the extraction efficiencies and the coverage at depth 30 (*p* = 0.5639, *r* = 0.08448). Overall, these results suggest that, using our protocol, the genome coverage is mildly affected by the SARS-CoV-2 concentration in the range covered here (from 1 × 10^4^ to 1 × 10^6^ cRNA/L), and highlight the adverse impact of RNA extract or wastewater sample freezing on the quality of sequencing data.

### ARTIC Multiplex PCR Efficiency

**FIGURE 4:**
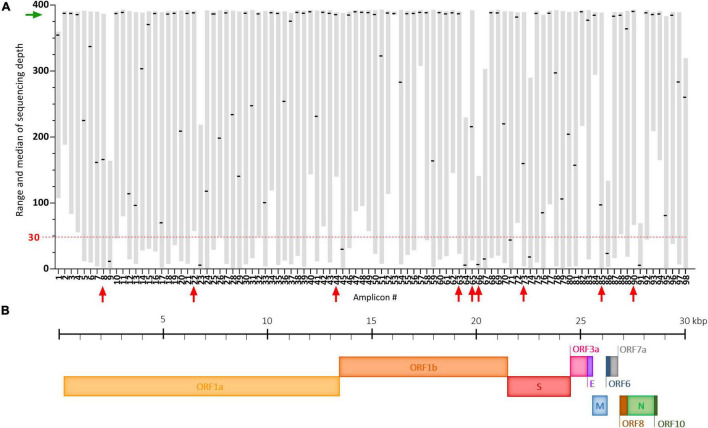
Coverage analysis of the SARS-CoV-2 genome using our adapted ARTIC sequencing protocol. **(A)** Plot depicting the range (gray floating bars) and medians (black horizontal lines) of sequencing depth obtained for each of the 98 amplicons of the ARTIC multiplex PCR in 35 raw wastewater samples included in the study. Very poorly covered amplicons (median < 30, red dashed line) are indicated by red arrows and the green arrow shows satisfying medians of sequencing depth. **(B)** Schematic representation of the SARS-CoV-2 genome.

**FIGURE 5:**
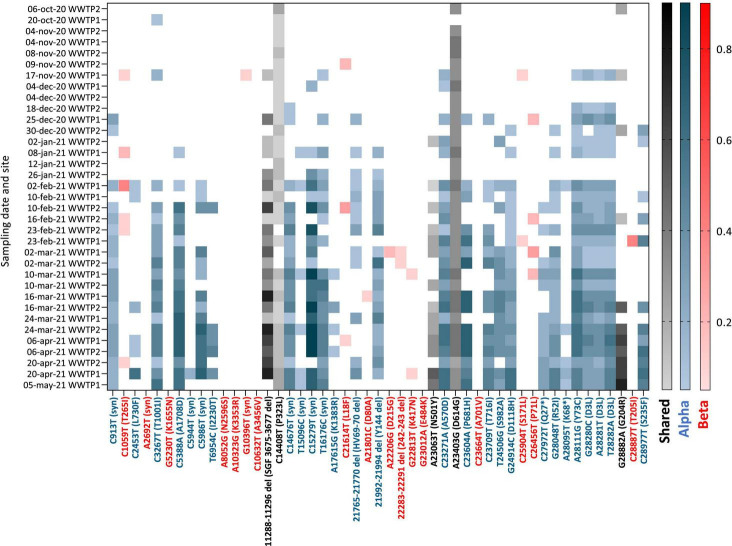
Heatmap of the frequency (color shades) of VOC signature mutations (*x* axis) in raw wastewater samples collected in 2 WWTP from Nantes overtime (*y* axis). Blue: Alpha VOC, red: Beta VOC and black: shared mutations. White indicates that no mutation was detected after applying quality filtering and detection thresholds.

The ARTIC multiplex PCR V3 creates 98 overlapping amplicons enabling amplification of the full SARS-CoV-2 genome, but with potential heterogeneous yields (Tyson et al., 2020). Here, using our adapted protocol, we observed that some of these amplicons were systematically very poorly covered despite good global genome coverage (Figure 4A). These dropouts (median of sequencing depth < 30) are amplicons #9, #23, #45, #64, #66, #67, #74, #86, and #91 and span regions summarized in Figure 4B and Supplementary Table 4. Potential mutations occurring in these regions could be missed following our protocol. Two already known mutations: A2692T (synonymous) carried by the Beta VOC and T6954C (I2230T) carried by the Alpha VOC, are covered by such amplicons (#9 and #23, respectively) (Supplementary Table 4). In our study of SARS-CoV-2 VOC circulation (Figure 5), the A2692T mutation was never detected but we managed to identify high frequency SNVs for the T6954C mutation in samples exhibiting the highest sequencing depths for the #23 amplicon (Figure 4A). Eventually, the vast majority of amplicons (91%) were sequenced at a median depth >30, enabling SARS-CoV-2 genome sequencing and the detection of most VOC signature mutations in the challenging wastewater matrix.

### Circulation of SARS-CoV-2 VOC in the City

Having adapted the sequencing protocol to fit to wastewater matrix, we carried on with the analysis of SARS-CoV-2 variants in samples collected between October 2020 and May 2021 from two WWTPs (WWTP1 and WWTP2) from the same city in western France. Of 45 WW samples submitted to the adapted sequencing protocol, we retained 35 with depth 30 coverage >70% (ranging from 74 to 100% with a median of 88%) for analysis of VOC circulation (Supplementary Table 1). Among those, 19 (54%) came from WWTP2 and 16 (46%) from WWTP1. Detected SNVs were analyzed and compared to signature mutations of the four VOC: Alpha, Beta, Gamma, and Delta (Supplementary Table 3). Their frequencies for each sample are plotted in Figure 5. Early SARS-CoV-2 mutations such as P323L and D614G were detected throughout the period analyzed and enabled method validation. We observed multiple signature mutations of the Alpha VOC accumulating over the analyzed period (Figure 5, blue). Importantly, oldest samples (October 2020 to early November 2020) exhibit none or only one mutation of the Alpha VOC, whereas more than 25 Alpha VOC specific mutations were detected for the most recent samples (April–May 2021). Most signature mutations were detected in combination as soon as early January 2021. Altogether, these data indicate that the Alpha VOC was introduced in the city during the analyzed period to finally become predominant, probably in early February 2021. Some signature mutations of the Beta VOC were detected sporadically, sometimes as combinations of 2–3 signature mutations for the same sample, but this was erratic over time and mutation frequencies remained low. These data are compatible with a weak circulation of the Beta VOC in the studied city during this period. Finally, we found no significant occurrence of Gamma and Delta variants signature mutations over the analyzed period.

### Tracking the Emergence of the Alpha VOC in the City

**FIGURE 6:**
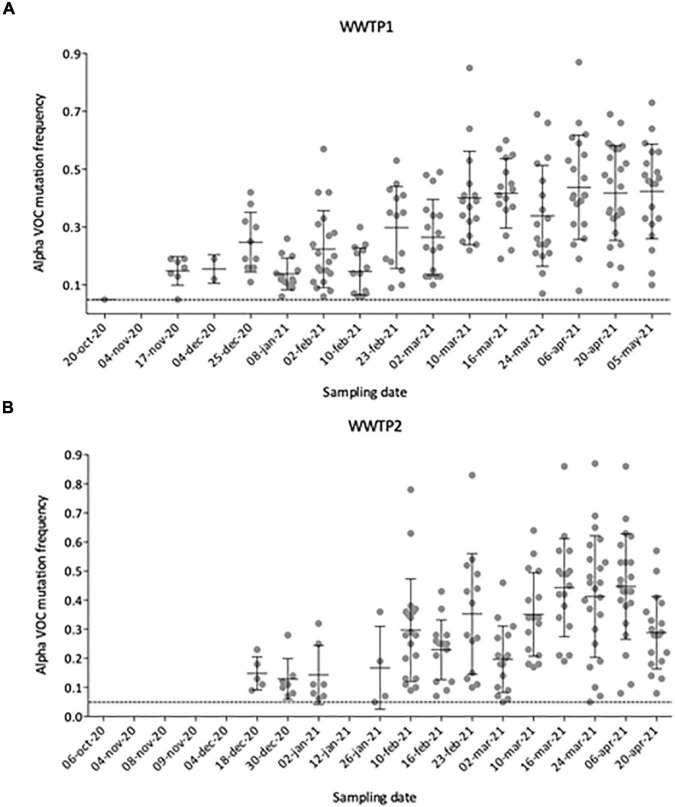
Number and individual frequencies (dots) of SARS-CoV-2 Alpha VOC over time in 35 raw wastewater samples from WWTP1 **(A)** and WWTP2 **(B)**, with median frequency (horizontal lines) and standard deviation (error bars). Only mutation with frequencies above 5% (dotted line) were considered.

To better define the date of the Alpha VOC introduction in the city, we plotted the number and frequency of detected Alpha VOC signature mutations throughout the analyzed period (Figures 6A,B). From October 2020 to early November 2020, no Alpha VOC signature mutation can be detected except one (20-Oct-20 in WWTP1), for which the frequency is just above our threshold of 5%. First co-occurrences of signature mutations appear in mid-November 2020 for WWTP1 and mid-December for WWTP2, with respectively, 8 and 5 Alpha VOC specific mutations at a median frequency <20%. From mid-November to the end of January, the number of Alpha VOC signature mutations tended to increase while the median frequencies remained around 20%. On two instances, Alpha VOC signature mutations were not detected. Finally, from February onward, the number of detected Alpha VOC signature mutations plateaued to its maximum of 20–25, while the median frequency increased to reach a maximum of 50% in April–May 2020. Individual mutation frequencies remained highly heterogeneous, varying from 10 to 85% in most samples.

Interestingly, some of these mutations being covered by the same amplicon, their presence in the same read was studied in samples corresponding to the introduction of the Alpha VOC. The three mutations responsible for the D3L substitution (28280-28282 GAT-CTA), highly specific of the Alpha VOC (see text footnote 1),were always found together on the same read for the following samples: 17-nov-20 WWTP1, 18-dec-20 WWTP2, 25-dec-20 WWTP1, and 30-dec-20 WWTP2 (Supplementary Figure 1). For the 25-dec-20 WWTP1 sample, the C23604A (P681H) and C23709T (T716I) mutations were found together in 35 out of 100 reads. Finally, for the 30-dec-20 WWTP2 sample, the G28048T (R52I) and A28111G (Y73C) mutations were identified together in 6 out of 307 reads and the G28882A (G204R) and C28977T (S235F) mutations in 21 out of 57 reads. These data show that viral strains with multiple signature mutations specific of the Alpha VOC circulated in the studied city as early as mid-November 2020.

**FIGURE 7:**
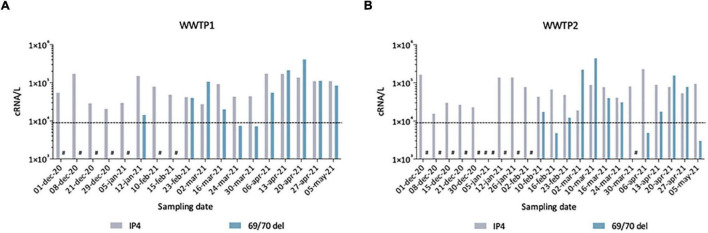
Quantification of total SARS-CoV-2 (IP4) and Alpha VOC (69/70 del) estimated by qRT-PCR in raw wastewater samples from WWTP1 **(A)** and WWTP2 **(B)**. Some samples gave signals below the theoretical limit of detection (LOD) of 9 × 10^3^ cRNA/L (dotted line), others gave no signal/no Ct (#).

To further validate our observations, we compared these sequencing results to quantitative data generated by two qRT-PCR assays, one SARS-CoV-2 generic qRT-PCR (IP4, see “Materials and Methods”) run on fresh samples and one specifically targeting the SΔ69/70, performed retrospectively to quantify the Alpha VOC (Figure 7). From December 2020 to May 2021, we detected SARS-CoV-2 genomes in wastewaters at around 10^4^–10^5^ cRNA/L. The first SΔ69/70 qRT-PCR positive results occurred on 12 January 2021 in WWTP1 (A) and 10 February 2021 in WWTP2 (B), with concentrations very close to the limit of detection (LOD). The SΔ69/70 was again detected on February 23 for both WWTPs at high levels (>10^5^ cRNA/L). For both WWTPs, we can see a decrease at the end of March 2021, followed by a progressive increase to reach a peak at the end of April 2021. The SΔ69/70 qRT-PCR results showed more fluctuations, and detected the Alpha VOC later, than the sequencing approach, but both techniques agree on the detection of the Alpha VOC by January 2021 in the studied city, first as a minority strain, and show a gradual replacement of the initial SARS-CoV-2 strain with the Alpha VOC.

## Discussion

Given the increasing prevalence of new SARS-CoV-2 variants, identifying VOC and monitoring their spread in the population is crucial. SARS-CoV-2 WGS has proven to be a substantial tool facilitating the understanding of COVID-19 outbreak transmission dynamics and the surveillance of viral genetic diversity (World Health Organisation [Who], 2020). To be efficient, clinical surveillance should rely on rapid and widespread PCR testing, along with a thorough SARS-CoV-2 WGS program. In most locations equipped with a sewage collection system, the use of Environmental Surveillance (ES), through wastewater sequencing of SARS-CoV-2 genomes, could contribute to achieve this goal in a timely and cost-effective manner compared to the individual-centered testing. Sewage samples also hold many advantages over clinical sampling considering collection is relatively easy, ethical issues and sampling bias (i.e., favoring severe cases) are limited, and only a few samples are needed to have a global picture of viral diversity in a community, including asymptomatic infections (Farkas et al., 2020; Michael-Kordatou et al., 2020). This has already been shown and used with other viruses (Lodder et al., 2012; Manor et al., 2014). Yet, they also represent a difficult matrix with a low viral concentration, hence requiring the adaptation of dedicated methods for efficient SARS-CoV-2 WGS.

Here we successfully adapted the SARS-CoV-2 sequencing technique described by the ARTIC Network (see text footnote 2) for clinical samples, to sewage samples. The ARTIC-400 multiplex PCR was shown to offer good performance with degraded or high Ct samples (Tyson et al., 2020), and thus appeared well suited for the complex wastewater matrix, considering that longer amplicons might be difficult to obtain from partially fragmented genomes (Wurtzer et al., 2021b). Since our study was conducted, others have shown that this primer scheme is indeed more efficient than others on raw influent wastewater (Lin et al., 2021). Here, compared to the published ARTIC protocol, changes were introduced at the RT, PCR and library preparation steps to increase the initially low genome coverage breadth and depth (Figure 1). To our knowledge, performing each pool of the ARTIC multiplex PCR in triplicates and pooling them was not reported in other studies and had a major impact here, with about a 2-fold increase of coverage breadth for a given depth. This confirms that the success of WGS protocols highly depends on the availability of enough high-quality genetic material to maximize sequencing yield and the soundness of sequence data. These changes are not specific to the wastewater matrix and may also be useful for sequencing SARS-CoV-2 from difficult matrices, when the viral load is low and/or the genome fragmented.

This adapted protocol allowed us to sequence SARS-CoV-2 genomes, with high coverage depth and breadth (>70% at >30×) despite low viral concentrations as measured by qRT-PCR. We observed a weak correlation between genome coverage and SARS-CoV-2 viral load, as previously reported by similar studies using ONT sequencing (Izquierdo-Lara et al., 2021; Lin et al., 2021). Of note, the application of the adapted method on stored samples to perform retrospective studies shed light on the adverse impact of freezing wastewater samples on the quality of sequencing data. Indeed, enveloped viruses like SARS-CoV-2 are commonly considered to be sensitive to freeze-thaw cycles. Besides, raw wastewater can contain detergent and other chemical products, which might contribute to disrupt the viral particles. Consequently, we recommend the use RNA extracted from fresh wastewater samples to perform SARS-CoV-2 quantification and sequencing according to the methods described in this study.

To efficiently monitor SARS-CoV-2 variants, methods should remain fast and affordable. Thus, we favored ONT sequencing, which is known for its lower entry and per base sequencing cost (compared to second generation sequencing technologies) and its ability to generate real-time data (Chang et al., 2020). Indeed, the method we describe here can provide information within 3 days of sewage collection, including the time for sample preparation, PCR, sequencing, data export and SNV analysis, for a cost of 55 € per sample (from RNA to sequence, using flow cells twice). This is higher than the previously reported 10£ (around 12 €) per clinical sample for ARTIC V3 (Tyson et al., 2020) but is still cost-efficient for epidemiological monitoring since sequencing SARS-CoV-2 genome from a WW sample gives information at a population level compared to an individual level for a clinical sample. Both time and price could be further reduced by bulk ordering of flow cells and reagents, additional adaptations of the library preparation, higher multiplexing and automated data analysis. Furthermore, the MinION sequencer is a portable device allowing on-field sequencing in WWTP on small series of samples, which may also contribute to reduce the time-to-result in some settings. However, one important limitation of ONT is its higher error rate when compared to second generation sequencing technologies (Chang et al., 2020). To ensure reliable identification of VOC, we applied stringent thresholds combining the per-base and per-read sequencing quality, breadth and depth of coverage (≥70% of genome at > 30 sequencing depth), SNV frequency (>5%) as well as, for indels, homopolymer length (<4). The absence of VOC signature mutations detection in oldest samples (October to early November 2020) confirms the validity of these thresholds. Of note, use of new flow cells with reduced error rate could allow reaching a deeper and broader coverage while reducing the thresholds to detect rare variants (R10.4, Oxford Nanopore Technologies, Oxford, United Kingdom).

Despite a satisfying production yield for a vast majority of the ARTIC amplicons, some regions were systematically absent or very poorly covered in our hands (Figure 4A). These amplicon dropouts are not news to the ARTIC Network, which already produced some work in order to fix this issue giving rise to the V3 primer scheme (Tyson et al., 2020). However, a study still reported #74 amplicon dropout with the V3 primer scheme, as in our study, and fixed the problem by adjusting concentration of its primer set (Pater et al., 2021). Other panels generating longer amplicons, designed for devices compatible with long-read sequencing such as the MinION, could also be considered. Yet, it is important to keep in mind that most studies testing these approaches were performed on clinical samples and may not reflect what occurs with wastewater samples in which targeted genomes can be fragmented and potentially not equally preserved. Indeed, we observed here dropouts for several amplicons aside #74 that may represent WW-specific problematic regions of the viral genome, because of lower stability or higher sensitivity to PCR inhibitors.

Frequent updates of the primer panel are also necessary to adapt to new variants and avoid under-detection of certain mutations, as achieved by recently by optimizing the ARTIC V4 version for sequencing the Delta VOC (Davis et al., 2021). Importantly, the V3 primer panel was recently used to sequence the Omicron VOC in an aircraft wastewater sample, suggesting that our method can still be applied in the frame of Omicron dominance (Ahmed et al., 2022). Future updates of the ARTIC-400 primer panels could be considered to further adapt our protocol to the current circulating variants.

Major consensus genotypes detected in wastewaters were previously found to be identical to clinical genomes from the same area and can identify the predominant virus strain circulating in a population (Crits-Christoph et al., 2021). However, this approach is not suitable to identify alternative genotypes in the population being studied, which constitutes the strength of wastewater-based sequencing. In addition, it results in artificial chimeric consensus genotypes that do not depict an actual virus (Izquierdo-Lara et al., 2021). Here, we made the choice to not generate consensus genomes with sequencing data obtained from sewage samples, and rather focused on the identification of SARS-CoV-2 VOC.

We show occurrences of Alpha VOC signature mutations at high frequencies and some Beta VOC signature mutations at low frequencies, while the Gamma and Delta VOC were not observed in WW collected in the studied city. This is consistent with the known circulation of these variants in France (Santé Publique France, 2021), where the Alpha VOC became predominant during the study period, while Beta and Gamma VOC remained rare. We observed three phases of the Alpha VOC spread (Figures 6A,B). In the first phase, characterized by a unique Alpha VOC signature mutation occurring at a very low frequency, we consider that the Alpha VOC was not detected. The third phase, starting in February 2021, can be confidently interpreted as the spread of the Alpha VOC, given the high number and frequencies of signature mutations and the documented circulation of this VOC in France at the time (Gaymard et al., 2021). The second phase, between mid-November and the end of January, combines fewer signature mutations with erratic detection. Indeed, a small number of Alpha VOC-specific mutations (Figure 6), not always the same (Figure 5), were detected at low frequencies. In WWTP2 especially, some mutations detected in late December 2020 or early January 2021 were no observed with samples from January 12th and 26th. This could be the early sign of the Alpha VOC clusters appearing and disappearing in the served population. Since it might also result from the co-circulation of multiple minority strains with independent mutations, we took advantage of ARTIC amplicons spanning several of these mutations, and confirmed that multiple reads bore couples or triads of signature mutations representing true haplotypes rather than independent, randomly co-occurring SNV. Together, our results strongly suggest that the Alpha VOC or closely related strains were introduced in the studied city during November 2020. This is in agreement with previous studies where SARS-CoV-2 was sequenced in WW samples. In the United Kingdom, Wilton and his co-workers were able to detect the Alpha VOC in WWs from London as early as November 2020 by nested-PCR amplification and sequencing of two regions of the Spike gene (Wilton et al., 2021). The Alpha VOC was also detected through WGS by mid-December in WW from Switzerland (Jahn et al., 2021), in December 2020 in Israel (Bar-Or et al., 2021) and in January 2021 in Nice, France (Rios et al., 2021).

In previous studies, the frequency of the Alpha VOC in SARS-CoV-2 strains infecting the population was estimated from those of signature mutations in WW data (Jahn et al., 2021; Rios et al., 2021; Wilton et al., 2021). Here, the frequencies of the different Alpha VOC signature mutations were highly heterogeneous, comprised between 5% (our threshold) and 85% with the median plateauing at 50%. This could be due in part to differences in amplification efficiencies, since mutations covered by the same amplicon often display similar frequencies (Figure 5). This also likely arose from our choice to consider all mutations known to be specific for the Alpha VOC lineage (Supplementary Table 3), even when they emerged later or only occurred in a fraction of these viruses, such as A28095T. Yet, some Alpha VOC signature mutations, known to occur in the whole lineage, were also less frequently detected than others, such as the 21765-70 and 21992-4 deletions, which was already shown in another study combining ARTIC-400 and ONT sequencing (Rios et al., 2021) and might be due to the sequencing approach. These biases, and possibly others, result in an underestimation of the actual magnitude of Alpha VOC frequency in the population when considering median frequencies of its mutations in WW.

An alternative to WGS, mutation-specific RT-PCR, was used in parallel to detect and quantify the Alpha VOC in our samples. Its design allows targeting the SΔ69-70 mutation, highly specific of the Alpha VOC at the time of the study, with a PCR efficiency and a limit of detection in the range of classical, pan-SARS-CoV-2 qRT-PCR sets (Wurtzer et al., 2021a). Here, SΔ69-70 results were more fluctuant and belated than sequencing data, with a first detection in January followed by weeks of absence of detection before the rapid increase in Alpha VOC concentration mid-February. Since confidence in high Ct values decreases, it is known that the error estimates increase at low virus concentrations (Polo et al., 2018) such as those observed for SARS-CoV-2 in wastewaters, especially for the Alpha VOC at the beginning of its spreading the population. Our data suggest that WGS of SARS-CoV-2 is more sensitive than mutation-specific qRT-PCR assays for detecting an emerging VOC, probably because it combines the detection of multiple signature mutations. It is also necessary to confirm the co-occurrence of several mutations as haplotypes, and conclusively identify a VOC. Yet, since VOC-specific qRT-PCR can provide faster and quantitative results (Wurtzer et al., 2021a) both approaches are complementary, each addressing specific needs and phases of VOC circulation (identification vs. spread).

Another important advantage of WGS is that SNV analysis can reveal mutations that were not previously observed in the global database and could also be used to monitor novel mutations. These newly observed mutations could (1) be the result of technical errors, like PCR mistakes or sequencing noise, (2) belong to minority (or even defective) genomes that are overlooked in clinical samples when the consensus sequence is generated, (3) be specific of the intestinal shedding of SARS-CoV-2 while most data are derived from nasopharyngeal swabs, (4) simply not persist in the population due to genetic drift or fitness disadvantage, (5) arise from non-human reservoirs also shedding into sewage (Smyth et al., 2022). Additional work on SARS-CoV-2 genetic diversity in different compartments of infected individuals, and in commensal animals, are still needed to better interpret the vast amount of information provided by WW sequencing. Yet, beside the monitoring of known VOC, this approach may contribute to discover novel viral mutations that are threatening for vaccine efficacy.

## Conclusion

Here, we described the successful adaptation of a SARS-CoV-2 whole-genome sequencing approach for wastewater samples. This technique has the advantages of being (1) time-efficient, providing sequencing data within 3–4 days of sewage samples arriving in the laboratory and (2) cost-efficient as it gives information at a community level, (3) reliable in a range of SARS-CoV-2 RNA concentration of 10^4^–10^5^ cRNA/L. Our study also underlines the value of wastewater-based SARS-CoV-2 WGS, which detected the circulation of the Alpha VOC in a French city earlier than a specific qRT-PCR, and identified shifts in variant predominance. Nevertheless, as multiple strains of SARS-CoV-2 are mixed in sewage samples, the sequencing approach in this matrix only detects mutations in association with a genome position instead of strains in association with an individual, providing indirect proof for the presence of a lineage. Therefore, thorough comparisons with clinical data are needed in order to identify the degree and limits to which environmental surveillance could be used as an early-detection tool to support public health decision-making. Within this frame, wastewater-based SARS-CoV-2 sequencing can contribute to monitor epidemiologically or clinically relevant mutations or variants within an unbiased population.

## Members of the Obepine Consortium

The steering committee of the scientific interest group Obépine is composed of Jean-Luc Bailly (Laboratoire Microorganismes, Génome et Environnement, LMGE, UMR 6023 CNRS-Université Clermont Auvergne, Clermont-Ferrand, France), Christophe Gantzer (Laboratoire de Chimie Physique et Microbiologie pour les Matériaux et l’Environnement, LCPME, UMR 7564 CNRS-Université de Lorraine, Nancy, France), Françoise S. Le Guyader (Ifremer Nantes, France), Yvon Maday (Institut Carnot Smiles, Sorbonne Université, Laboratoire Jacques-Louis Lions, UMR 7598 and Institut Universitaire de France), Vincent Maréchal (Sorbonne Université, INSERM, Centre de Recherche Saint-Antoine, 75012, Paris, France), Jean-Marie Mouchel (Sorbonne Université, CNRS, EPHE, UMR 7619 Metis, Paris, France, e-LTER Zone Atelier Seine), Laurent Moulin (Laboratoire R&D, Eau de Paris, France), Remy Teyssou (Val de Grâce, Unité de Virologie de l’IRBA. Institut de Recherche Biomédicale des Armées, Brétigny, France), Sébastien Wurtzer (Laboratoire R&D, Eau de Paris, France).

## Data Availability Statement

The datasets presented in this study can be found in the online repository DataRef from the SeaDataCloud Network: https://data-dataref.ifremer.fr/bioinfo/ifremer/obepine/lsem/data/dna-sequence-raw/, 20210210-run-1, 20210303-run-2, 20210324-run-3, 20210413-run-4, 20210505-run-5, 20210507-run-6, 20210708-run-7, and 20210727-run-8.

## Author Contributions

LB and MD conceived and designed the study and wrote the manuscript. JS, SJ, and LB performed the experiments. SW and LM designed the Alpha VOC qRT-PCR assay. LB, JS, AB, and MD analyzed the data. MD and FL obtained the funding. LB, JS, AB, SW, LM, FL, MD, and the OBEPINE Consortium reviewed the manuscript. All authors contributed to the article and approved the submitted version.

## Conflict of Interest

The authors declare that the research was conducted in the absence of any commercial or financial relationships that could be construed as a potential conflict of interest.

## Publisher’s Note

All claims expressed in this article are solely those of the authors and do not necessarily represent those of their affiliated organizations, or those of the publisher, the editors and the reviewers. Any product that may be evaluated in this article, or claim that may be made by its manufacturer, is not guaranteed or endorsed by the publisher.

## Footnotes

1
https://nextstrain.org

2
https://artic.network/ncov-2019
