# Supplementary material for: SARS-CoV-2 Whole-Genome Sequencing Using Oxford Nanopore Technology for Variant Monitoring in Wastewaters
Source: Front Microbiol. 2022 Jun 9;13:889811. doi: 10.3389/fmicb.2022.889811 (PMC9218694; doi:10.3389/fmicb.2022.889811)

**Supplementary information**

# SARS-CoV-2 whole-genome sequencing using Oxford Nanopore Technology for variant monitoring in wastewaters

**Authors’ names**

Laure Barbé^a^, Julien Schaeffer^a^, Alban Besnard^a^, Sarah Jousse^a^, Sébastien Wurtzer^b^, Laurent Moulin^b^, Scientific Interest Group Obépine#, Françoise S Le Guyader^a^ and Marion Desdouits*^a^

## Supplementary Table S1. Characteristics of sequenced samples

| **Sampling site** | **Sampling date** | **Extraction efficiency (%)** | **Ct IP4** | **Total SARS-CoV-2 (cRNA / L)** | **Ct del69/70** | **Alpha VOC (cRNA / L)** | **Optimization line** | **Run #** | **Initial sample** | **Storage period** | **Depth 30 coverage (%)** |
| --- | --- | --- | --- | --- | --- | --- | --- | --- | --- | --- | --- |
| WWTP1 | 14-jul-20 | 1.6 | 36.0 | 3.8 x 10^4^ | ND | ND | 1 | 1 | Frozen RNA | 7 months | 0 |
| WWTP1 | 20-oct-20 | 1.0 | 33.4 | 2.0 x 10^5^ | ND | ND | 1 | 1 | Frozen RNA | 4 months | 25 |
| WWTP1 | 20-oct-20 | 4.0 | 33.6 | 6.9 x 10^4^ | ND | ND | 1 | 1 | Frozen WW | 4 months | 4 |
| WWTP1 | 20-oct-20 | 5.0 | 36.1 | 6.5 x 10^4^ | ND | ND | 1 | 1 | Frozen WW | 4 months | 3 |
| WWTP2 | 04-nov-20 | 7.1 | 32.6 | 3.1 x 10^5^ | ND | ND | 1 | 1 | Frozen RNA | 3 months | 16 |
| WWTP2 | 04-nov-20 | 27.0 | 31.7 | 2.5 x 10^5^ | ND | ND | 1 | 1 | Frozen WW | 3 months | 84 |
| WWTP2 | 04-nov-20 | 14.0 | 33.0 | 4.8 x 10^5^ | ND | ND | 1 | 1 | Frozen WW | 3 months | 64 |
| WWTP2 | 08-nov-20 | 4.9 | 31.4 | 6.5 x 10^5^ | ND | ND | 1 | 1 | Frozen RNA | 3 months | 3 |
| WWTP1 | 02-feb-21 | 7.0 | 35.4 | 5.6 x 10^4^ | ND | ND | 1 | 1 | Frozen RNA | 6 days | 0 |
| WWTP1 | 02-feb-21 | 0 | 37.0 | 9.0 x 10^4^ | ND | ND | 1 | 1 | Frozen RNA | 6 days | 0 |
| WWTP1 | 02-feb-21 | 0 | 36.3 | 8.0 x 10^3^* | ND | ND | 1 | 1 | Frozen RNA | 6 days | 2 |
| WWTP1 | 15-feb-21 | 15.6 | 35.6 | 4.9 x 10^4^ | No Ct | 0 | 2 | 2 | Frozen RNA | 2 weeks | 16 |
| WWTP1 | 15-feb-21 | 1.0 | 35.0 | 4.1 x 10^4^ | ND | ND | 2 | 2 | Frozen RNA | 2 weeks | 54 |
| WWTP1 | 15-feb-21 | 0 | 36.6 | 3.1 x 10^4^ | ND | ND | 2 | 2 | Frozen RNA | 2 weeks | 10 |
| WWTP1 | 15-feb-21 | 0 | 34.3 | 4.4 x 10^3^* | ND | ND | 2 | 2 | Frozen RNA | 2 weeks | 49 |
| WWTP2 | 16-feb-21 | 5.5 | 35.1 | 6.7 x 10^4^ | 42.0 | 4.9 x 10^3^* | 2 | 2 | Frozen RNA | 2 weeks | 37 |
| WWTP2 | 16-feb-21 | 1.0 | 34.8 | 3.3 x 10^4^ | ND | ND | 2 | 2 | Frozen RNA | 2 weeks | 31 |
| WWTP2 | 16-feb-21 | 2.0 | 35.2 | 3.4 x 10^4^ | ND | ND | 2 | 2 | Frozen RNA | 2 weeks | 36 |
| WWTP2 | 16-feb-21 | 0 | 34.3 | 4.6 x 10^3^* | ND | ND | 2 | 2 | Frozen RNA | 2 weeks | 39 |
| WWTP2 | 23-feb-21 | 9.7 | 35.6 | 4.9 x 10^4^ | 40.5 | 1.3 x 10^4^ | 3 | 3 | Frozen RNA | 1 month | 75 |
| WWTP2 | 23-feb-21 | 4.0 | 35.5 | 4.9 x 10^4^ | ND | ND | 3 | 3 | Frozen RNA | 1 month | 91 |
| WWTP1 | 23-feb-21 | 11.0 | 35.8 | 4.2 x 10^4^ | 38.6 | 4.2 x 10^4^ | 3 | 3 | Frozen RNA | 1 month | 84 |
| WWTP1 | 23-feb-21 | 4.0 | 35.4 | 9.1 x 10^4^ | ND | ND | 3 | 3 | Frozen RNA | 1 month | 93 |
| WWTP1 | 02-mar-21 | 1.3 | 36.5 | 2.8 x 10^4^ | 37.1 | 1.1 x 10^5^ | 3 | 3 | Frozen RNA | 3 weeks | 80 |
| WWTP1 | 02-mar-21 | 16.0 | 35.0 | 2.7 x 10^4^ | ND | ND | 3 | 3 | Frozen RNA | 3 weeks | 88 |
| WWTP2 | 02-mar-21 | 1.7 | 37.1 | 1.9 x 10^4^ | 36.0 | 2.3 x 10^5^ | 3 | 3 | Frozen RNA | 3 weeks | 81 |
| WWTP2 | 02-mar-21 | 47.0 | 35.2 | 9.8 x 10^4^ | ND | ND | 3 | 3 | Frozen RNA | 3 weeks | 88 |
| WWTP1 | 10-mar-21 | 4.9 | 34.7 | 8.5 x 10^4^ | ND | ND | 3 | 3 | Frozen RNA | 1 week | 82 |
| WWTP2 | 10-mar-21 | 4.3 | 34.6 | 8.9 x 10^4^ | 34.9 | 4.6 x 10^5^ | 3 | 3 | Frozen RNA | 1 week | 85 |
| WWTP1 | 16-mar-21 | 14.6 | 34.5 | 9.3 x 10^4^ | 39.7 | 2.1 x 10^4^ | 3 | 3 | Frozen RNA | 1 week | 85 |
| WWTP2 | 16-mar-21 | 10.1 | 34.9 | 7.8 x 10^4^ | 38.7 | 4.2 x 10^4^ | 3 | 3 | Frozen RNA | 1 week | 86 |
| WWTP1 | 24-mar-21 | 5.2 | 35.8 | 4.4 x 10^4^ | 41.3 | 7.5 x 10^3^* | 4 | 4 | Fresh RNA | 2 weeks | 51 |
| WWTP2 | 24-mar-21 | 2.4 | 35.9 | 4.1 x 10^4^ | 39.0 | 3.3 x 10^4^ | 4 | 4 | Fresh RNA | 2 weeks | 100 |
| WWTP3 | 28-mar-21 | 10.9 | 33.5 | 1.8 x 10^5^ | 39.3 | 2.7 x 10^4^ | 4 | 4 | Fresh RNA | 1 week | 93 |
| WWTP3 | 01-apr-21 | 18.1 | 34.1 | 1.3 x 10^5^ | 35.8 | 2.6 x 10^5^ | 4 | 4 | Fresh RNA | 1 week | 95 |
| WWTP3 | 06-apr-21 | 7.1 | 33.1 | 2.3 x 10^5^ | 34.6 | 5.8 x 10^5^ | 4 | 4 | Fresh RNA | 5 days | 91 |
| WWTP1 | 06-apr-21 | 2.4 | 33.5 | 1.7 x 10^5^ | 38.2 | 5.8 x 10^4^ | 4 | 4 | Fresh RNA | 5 days | 98 |
| WWTP2 | 06-apr-21 | 2.0 | 33.1 | 2.3 x 10^5^ | 42.0 | 5.0 x 10^3^* | 4 | 4 | Fresh RNA | 5 days | 97 |
| WWTP3 | 08-apr-21 | 5.2 | 34.9 | 7.5 x 10^4^ | 38.0 | 6.5 x 10^4^ | 4 | 4 | Fresh RNA | 3 days | 98 |
| WWTP1 | 14-jul-20 | 1.6 | 36.0 | 3.8 x 10^4^ | ND | ND | 4 | 5 | Frozen RNA | 10 months | 25 |
| WWTP1 | 20-oct-20 | 1.0 | 33.4 | 2.0 x 10^5^ | ND | ND | 4 | 5 | Frozen RNA | 7 months | 89 |
| WWTP1 | 04-nov-20 | 10.9 | 33.1 | 2.3 x 10^5^ | ND | ND | 4 | 5 | Frozen RNA | 6 months | 96 |
| WWTP1 | 17-nov-20 | 13.8 | 34.1 | 1.2 x 10^5^ | ND | ND | 4 | 5 | Frozen RNA | 6 months | 93 |
| WWTP1 | 24-nov-20 | 9.8 | 36.0 | 3.8 x 10^4^ | ND | ND | 4 | 5 | Frozen RNA | 6 months | 24 |
| WWTP1 | 04-dec-20 | 21.6 | 35.4 | 5.5 x 10^4^ | No Ct | 0 | 4 | 5 | Frozen RNA | 5 months | 97 |
| WWTP1 | 25-dec-20 | 14.0 | 36.4 | 2.9 x 10^4^ | No Ct | 0 | 4 | 5 | Frozen RNA | 4 months | 84 |
| WWTP1 | 08-jan-21 | 23.5 | 33.8 | 1.5 x 10^5^ | 40.2 | 1.5 x 10^4^ | 4 | 5 | Frozen RNA | 4 months | 94 |
| WWTP1 | 02-feb-21 | 7.0 | 35.4 | 5.6 x 10^4^ | ND | ND | 4 | 5 | Frozen RNA | 4 months | 90 |
| WWTP1 | 10-feb-21 | 15.4 | 34.8 | 8.0 x 10^4^ | No Ct | 0 | 4 | 5 | Frozen RNA | 3 months | 91 |
| WWTP1 | 23-feb-21 | 11.0 | 35.8 | 4.2 x 10^4^ | 38.6 | 4.2 x 10^4^ | 4 | 5 | Frozen RNA | 3 months | 85 |
| WWTP1 | 16-mar-21 | 14.6 | 34.5 | 9.3 x 10^4^ | 39.7 | 2.1 x 10^4^ | 4 | 5 | Frozen RNA | 2 months | 71 |
| WWTP1 | 24-mar-21 | 5.2 | 35.8 | 4.4 x 10^4^ | 41.3 | 7.5 x 10^3^* | 4 | 6 | Frozen RNA | 2 months | 83 |
| WWTP2 | 04-nov-20 | 7.1 | 32.6 | 3.1 x 10^5^ | ND | ND | 4 | 6 | Frozen RNA | 6 months | 90 |
| WWTP2 | 09-nov-20 | 8.0 | 31.7 | 5.7 x 10^5^ | ND | ND | 4 | 6 | Frozen RNA | 6 months | 95 |
| WWTP2 | 04-dec-20 | 5.1 | 37.4 | 1.6 x 10^4^ | No Ct | 0 | 4 | 6 | Frozen RNA | 5 months | 90 |
| WWTP2 | 18-dec-20 | 10.9 | 36.6 | 2.7 x 10^4^ | No Ct | 0 | 4 | 6 | Frozen RNA | 5 months | 82 |
| WWTP2 | 02-jan-21 | 3.6 | 36.8 | 2.3 x 10^4^ | No Ct | 0 | 4 | 6 | Frozen RNA | 4 months | 89 |
| WWTP2 | 19-jan-21 | 14.2 | 33.9 | 1.4 x 10^5^ | No Ct | 0 | 4 | 6 | Frozen RNA | 4 months | 91 |
| WWTP2 | 10-feb-21 | 23.8 | 35.8 | 4.3 x 10^4^ | 40.0 | 1.8 x 10^4^ | 4 | 6 | Frozen RNA | 3 months | 93 |
| WWTP2 | 16-feb-21 | 5.5 | 35.1 | 6.7 x 10^4^ | 42.0 | 4.9 x 10^3^* | 4 | 6 | Frozen RNA | 3 months | 80 |
| WWTP2 | 02-mar-21 | 1.7 | 37.1 | 1.9 x 10^4^ | 36.0 | 2.3 x 10^5^ | 4 | 6 | Frozen RNA | 2 months | 85 |
| WWTP2 | 16-mar-21 | 10.1 | 34.9 | 7.8 x 10^4^ | 38.7 | 4.2 x 10^4^ | 4 | 6 | Frozen RNA | 2 months | 86 |
| WWTP2 | 24-mar-21 | 2.4 | 35.9 | 4.1 x 10^4^ | 39.0 | 3.3 x 10^4^ | 4 | 6 | Frozen RNA | 2 months | 77 |
| WWTP2 | 09-jun-20 | 15.0 | 36.5 | 2.8 x 10^4^ | ND | ND | 4 | 7 | Frozen WW | >1 year | 3 |
| WWTP2 | 04-aug-20 | 14.0 | 37.3 | 1.7 x 10^4^ | ND | ND | 4 | 7 | Frozen WW | 10 months | 45 |
| WWTP2 | 06-oct-20 | 4.1 | 34.4 | 1.0 x 10^5^ | ND | ND | 4 | 7 | Frozen WW | 8 months | 74 |
| WWTP2 | 08-nov-20 | 4.9 | 31.4 | 6.5 x 10^5^ | ND | ND | 4 | 7 | Frozen WW | 7 months | 100 |
| WWTP2 | 30-dec-20 | 3.6 | 36.8 | 2.3 x 10^4^ | No Ct | 0 | 4 | 7 | Frozen WW | 6 months | 81 |
| WWTP2 | 12-jan-21 | 40.8 | 33.9 | 1.4 x 10^5^ | No Ct | 0 | 4 | 7 | Frozen WW | 5 months | 75 |
| WWTP2 | 26-jan-21 | 14.2 | 33.9 | 1.4 x 10^5^ | No Ct | 0 | 4 | 7 | Frozen WW | 5 months | 88 |
| WWTP2 | 20-apr-21 | 3.3 | 34.8 | 7.9 x 10^4^ | 36.5 | 1.6 x 10^5^ | 4 | 7 | Frozen RNA | 2 months | 75 |
| WWTP2 | 05-may-21 | 4.1 | 34.5 | 9.5 x 10^4^ | 42.7* | 3.1 x 10^3^* | 4 | 7 | Frozen RNA | 1 month | 63 |
| WWTP2 | 08-apr-20 | 2.7 | 36.2 | 3.4 x 10^4^ | ND | ND | 4 | 7 | Frozen WW | >1 year | 8 |
| WWTP2 | 08-apr-20 | 2.7 | 36.2 | 3.4 x 10^4^ | ND | ND | 4 | 8 | Frozen WW | >1 year | 17 |
| WWTP1 | 09-jun-20 | 20.1 | 36.4 | 3.0 x 10^4^ | ND | ND | 4 | 8 | Frozen WW | >1 year | 55 |
| WWTP1 | 04-aug-20 | 9.0 | 37.6 | 1.4 x 10^4^ | ND | ND | 4 | 8 | Frozen WW | 10 months | 55 |
| WWTP1 | 06-oct-20 | 7.6 | 33.8 | 1.5 x 10^5^ | ND | ND | 4 | 8 | Frozen WW | 8 months | 65 |
| WWTP1 | 24-nov-20 | 9.8 | 36.0 | 3.8 x 10^4^ | ND | ND | 4 | 8 | Frozen WW | 7 months | 82 |
| WWTP1 | 08-dec-20 | 6.8 | 33.6 | 1.7 x 10^5^ | No Ct | 0 | 4 | 8 | Frozen WW | 7 months | 42 |
| WWTP1 | 05-jan-21 | 3.8 | 36.4 | 3.0 x 10^4^ | No Ct | 0 | 4 | 8 | Frozen WW | 5 months | 80 |

*WWTP3 corresponds to a wastewater-treatment plant from which samples were used to optimize the sequencing process but no following study was performed.*

*Ct indicated are the mean of three experimental Ct.*

*ND: Not determined*

** Below limit of detection (9.0 x 10^3^ cRNA / L).*

*Fresh RNA was stored at 4°C whereas frozen WW and RNA were stored at -20°C.*

## Supplementary Table S2. PCR primer and probe sequences

| **Target** | **Oligonucleotide name** | **Sequence (5’ to 3’)** | **Concentration (nM)** | **Ref.** |
| --- | --- | --- | --- | --- |
| *Spike* | DEL69-70_F186 | TACTTGGTTCCATGCTATCT | 300 | 1 |
|  | DEL69-70_R340 | ACTGGGTCTTCGAATCTAA | 600 |  |
|  | DEL69-70_Ps307 | AGAAGTCTAACATAATAAGAGGCTGGA | 200 |  |
| *RdRp* | nCoV_IP4-14059Fw | GGTAACTGGTATGATTTCG | 400 | 2 |
|  | nCoV_IP4-14146Rv | CTGGTCAAGGTTAATATAGG | 400 |  |
|  | nCoV_IP4-14084Probe | TCATACAAACCACGCCAGG | 160 |  |

*^1^ R&D Laboratory, DRDQE, Eau de Paris, Ivry/Seine, France.*

*^2^ National Reference Center for Respiratory Viruses, Institut Pasteur, Paris, France.*

## Supplementary Table S3. Investigated mutations for Alpha, Beta, Gamma and Delta Variants of Concern (VOC)

| **Gene** | **Position** | **Ref** | **Alt** | **Amino acid mutation** | **VOC** |
| --- | --- | --- | --- | --- | --- |
| ORF1a | 913 | C | T | synonymous | Alpha |
| ORF1a | 1059 | C | T | T265I | Beta |
| ORF1a | 2453 | C | T | L730F | Alpha |
| ORF1a | 2692 | A | T | synonymous | Beta |
| ORF1a | 3267 | C | T | T1001I | Alpha |
| ORF1a | 5230 | G | T | K1655N | Beta |
| ORF1a | 5388 | C | A | A1708D | Alpha |
| ORF1a | 5944 | C | T | synonymous | Alpha |
| ORF1a | 5986 | C | T | synonymous | Alpha |
| ORF1a | 6954 | T | C | I2230T | Alpha |
| ORF1a | 10029 | C | T | T3255I | Delta |
| ORF1a | 10323 | A | G | K3353R | Beta |
| ORF1a | 11288-11296 | TCTGGTTTT | - | SGF3675-3677 Δ | Alpha, Beta, Gamma |
| ORF1b | 14408 | C | T | P314L | Alpha, Beta, Gamma, Delta |
| ORF1b | 14676 | C | T | synonymous | Alpha |
| ORF1b | 15096 | T | C | synonymous | Alpha |
| ORF1b | 15279 | C | T | synonymous | Alpha |
| ORF1b | 16176 | T | C | synonymous | Alpha |
| ORF1b | 17615 | A | G | K1383R | Alpha |
| S | 21614 | C | T | L18F | Beta, Gamma |
| S | 21618 | C | G | T19R | Delta |
| S | 21621 | C | A | T20N | Gamma |
| S | 21638 | C | T | P26S | Gamma |
| S | 21765-21770 | TACATG | - | HV69-70 Δ | Alpha |
| S | 21801 | A | C | D80A | Beta |
| S | 21846 | C | T | T95I | Delta |
| S | 21974 | G | T | D138Y | Gamma |
| S | 21987 | G | A | G142D | Delta |
| S | 21992-21994 | TAT | - | Y144 Δ | Alpha |
| S | 22029-22034 | AGTTCA | - | EF156-157 Δ | Delta |
| S | 22132 | G | T | R190S | Gamma |
| S | 22206 | A | G | D215G | Beta |
| S | 22283-22291 | TTACTTGCT | - | LLA241-243 Δ | Beta |
| S | 22812 | A | C | K417T | Gamma |
| S | 22813 | G | T | K417N | Beta |
| S | 22917 | T | G | L452R | Delta |
| S | 22995 | C | A | T478K | Delta |
| S | 23012 | G | A | E484K | Beta, Gamma |
| S | 23012 | G | C | E484Q | Delta |
| S | 23063 | A | T | N501Y | Alpha, Beta, Gamma |
| S | 23271 | C | A | A570D | Alpha |
| S | 23403 | A | G | D614G | Alpha, Beta, Gamma, Delta |
| S | 23525 | C | T | H655Y | Gamma |
| S | 23604 | C | A | P681H | Alpha |
| S | 23604 | C | G | P681R | Delta |
| S | 23664 | C | T | A701V | Beta |
| S | 23709 | C | T | T716I | Alpha |
| S | 24410 | G | A | D950N | Delta |
| S | 24506 | T | G | S982A | Alpha |
| S | 24642 | C | T | T1027I | Gamma |
| **Gene** | **Position** | **Ref** | **Alt** | **Amino acid mutation** | **VOC** |
| S | 24914 | G | C | D1118H | Alpha |
| S | 25088 | G | T | V1176F | Gamma |
| ORF3a | 25904 | C | T | S171L | Beta |
| E | 26456 | C | T | P71L | Beta |
| ORF8 | 27972 | C | T | Q27* | Alpha |
| ORF8 | 28048 | G | T | R52I | Alpha |
| ORF8 | 28095 | A | T | K68* | Alpha |
| ORF8 | 28111 | A | G | Y73C | Alpha |
| N | 28280-28282 | GAT | CTA | D3L | Alpha |
| N | 28882 | G | A | G204R | Alpha, Gamma |
| N | 28887 | C | T | T205I | Beta |
| N | 28977 | C | T | S235F | Alpha |

## Supplementary Table S4. ARTIC amplicons with lower depth of coverage

| **Amplicon #** | **Covered region** | **Median depth** | **Covered mutation / Corresponding VOC** | | |
| --- | --- | --- | --- | --- | --- |
| 9 | 2 592 to 2 827 bp | 12 | A2692T (synonymous) / Beta VOC | | |
| 23 | 6 873 to 7 036 bp | 6 | T6954C (I2230T) / Alpha VOC | | |
| 45 | 13 400 to 13 600 bp | 30 | - | | |
| 64 | 19 297 to 19 549 bp | 6 | - | | |
| 66 | 19 939 to 20 173 bp | 7 | - | | |
| 67 | 20 255 to 20 473 bp | 15 | - | | |
| 74 | 22 346 to 22 517 bp | 18 | - | | |
| 86 | 25 994 to 26 198 bp | 23 | - | | |
| 91 | 27 533 to 27 785 bp | 6 | - | | |
|  | |  | |  |  |


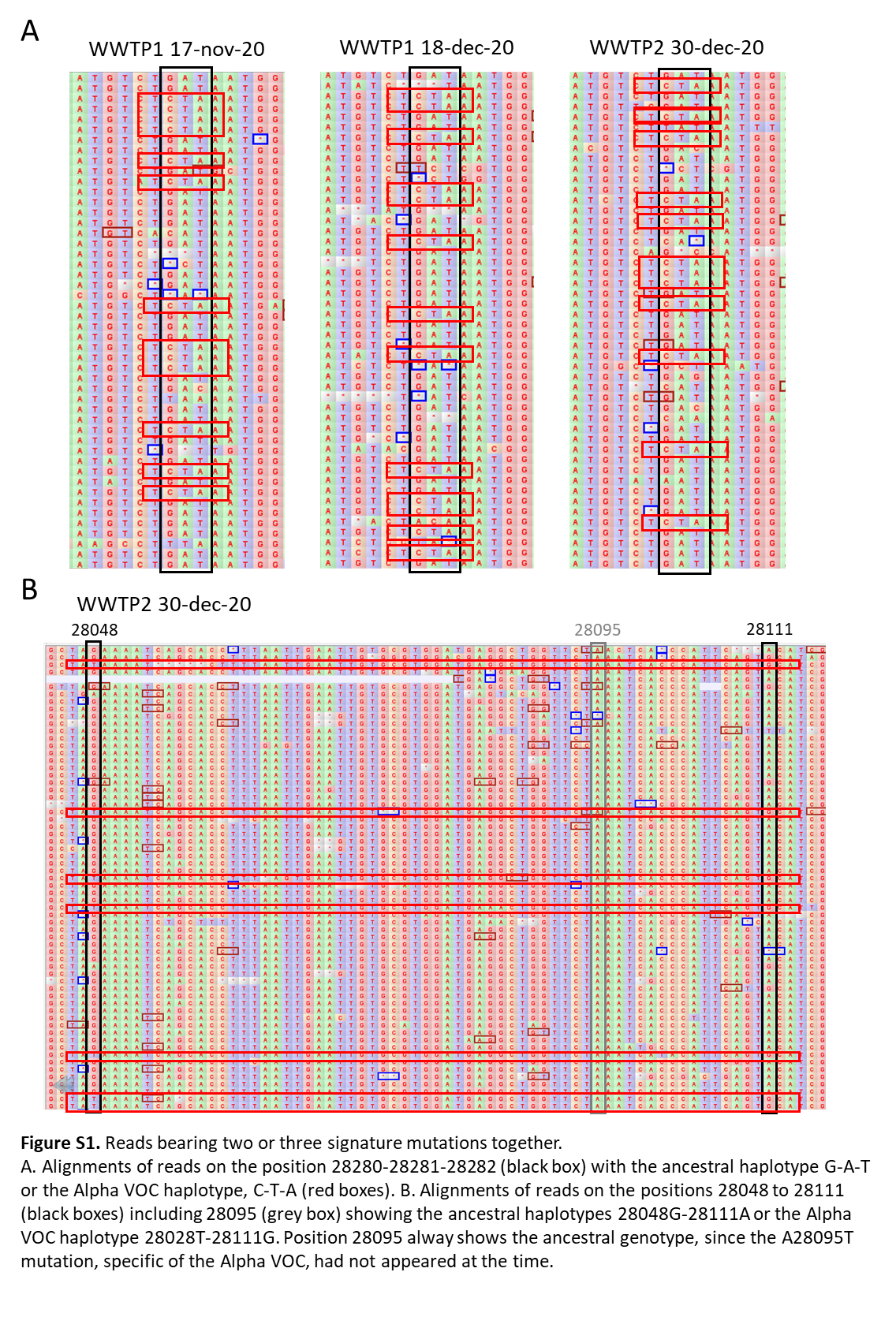

Supplement: Supplementary file 1 [file Table_1.DOCX]
